# Assessment of perceived health status among primary care patients in Southern Italy: findings from a cross-sectional survey

**DOI:** 10.1186/1477-7525-11-93

**Published:** 2013-06-10

**Authors:** Benedetto Manuti, Paolo Rizza, Claudia Pileggi, Aida Bianco, Maria Pavia

**Affiliations:** 1 Department of Health Sciences, Medical School, University of Catanzaro “Magna Græcia”, Catanzaro, Italy

**Keywords:** Quality of life, Mental health, Physical function, Health policy

## Abstract

**Background:**

The primary aim of this study was to measure HRQOL of primary care patients in one of the poorest areas of Italy, using SF-12, whereas the secondary aim was to identify subgroups of this population, according to socio-demographics, clinical characteristics, behavioural risk factors, and health services utilization, that manifest poorer HRQOL. These data may be helpful to policy makers to implement health care policies and social interventions for improving HRQOL.

**Methods:**

A cross-sectional survey was conducted in Southern Italy on primary care patients aged 18 and over. SF-12 was used to measure perceived health status. Physical component and mental summary scores were obtained. We performed univariate and multivariate analysis to evaluate eventual significant differences of mean PCS-12 and MCS-12 according to various characteristics (demographics, presence of chronic diseases, behavioral risk factors, and utilization of health services).

**Results:**

Of the 1467 participating in our survey, more than one third evaluated their health as unsatisfactory, noted significant limitations and reported problems on all SF-12-scales. Physical and mental summary scores showed an overall mean of 45.9 (SD ± 10.8) and 44.9 (SD ± 11.6), respectively. Statistical analysis showed significant differences in perceived health status by socio-demographic characteristics, such as gender, age, education level and employment status, by behavioral risk factors, chronic diseases and health services utilization.

**Conclusions:**

Our findings seem to indicate that primary care patients in Southern Italy have a poor HRQOL and this perception is even poorer in subgroups of the population, according to several sociodemographic, clinical characteristics, and behavioural risk factors. These results may have significant implications for health care policymakers, since they emphasize the need of developing effective and targeted strategies to improve HRQOL in Southern Italy.

## Background

Attention to perceived health status or “health related quality of life” (HRQOL) assessment has been increasing, since it represents an important component and one of the most effective strategies to evaluate quality of life, a multidimensional construct comprising physical, mental, social and economic components [1].

Many instruments are currently available to measure HRQOL, and Short-Form-12 Health Survey (SF-12) [2], is the most commonly used one. SF-12 is a short form health survey, developed as an alternative to the SF-36 [3]. SF-12 has been adopted by numerous health care delivery organizations, including the National Commission on Quality Assurance, and has been successfully tested in several countries, including Italy [4-7]. It has been extensively used in studies involving the general population [5,8,9], as well as vulnerable populations, such as elderly, minority subjects and patients with common diseases [10-14].

Several studies have focused on the distribution of socio-demographics, clinical characteristics, behavioural risk factors, and health services utilization according to physical and mental perceived health status, and have found significant differences associated with patients’ characteristics, such as gender, age, education level, social relationships, presence of medical problems, race or ethnic group, socio-economic and employment status [8,9,15-20].

During the 1990s Italy promoted political and financial regional decentralization, the consequence of which resulted in dramatic new responsibilities for regulating, planning and organizing health care delivery that has been transferred to the regions. The process of decentralization certainly represents progress but may increase risk of reducing HRQOL in poorer regions of South. Therefore, it is crucial to have regional disaggregated data on HRQOL, to identify possible differences among regions and with other countries and to assess the relationships between subjects’ characteristics and HRQOL to understand how policy makers can face deregulation and improve strategies to reach disadvantaged groups.

In Italy, all residents are provided a primary care physician (PCP) within the National Health Service, who acts as a “gate keeper” to preventive, diagnostic, therapeutic and rehabilitative services. Therefore, subjects attending PCPs certainly represent a subgroup of individuals seeking a health demand, and consequently of great interest in the assessment of HRQOL.

The primary aim of this study was to measure HRQOL of primary care patients in one of the poorest areas of Italy, using SF-12, whereas the secondary aim was to identify subgroups of this population, according to socio-demographics, clinical characteristics, behavioural risk factors, and health services utilization, that manifest poorer HRQOL. These data may be helpful to policy makers to implement health care policies and social interventions for improving HRQOL.

## Methods

### Study population and sample size

This cross sectional study was carried out in Calabria Region (Southern Italy), having a population of approximately 2 million living in 3 major cities and several smaller centers. About 1500 PCPs provide primary care to 1.6 million of patients (adults aged 18 years and over).

To enable the sample to better represent the patients characteristics, a multi-stage sampling was performed. First, we selected two cities having urban and demographic characteristics typical of the Southern part of the country; the former (Catanzaro) is the capital of the region and have 60 PCPs with 77,022 primary care patients; the latter (Crotone) is a small town with 38 PCPs (44,047 primary care patients). Then, we proportionally selected by simple random sampling 20 PCPs from the lists provided by the Local Health Units: 12 from Catanzaro and 8 from Crotone.

All consecutive subjects attending PCP for non urgent issues were recruited. Anonymous questionnaires, pre-tested on a sample of patients to ensure clarity of interpretation and ease of completion, were administered before the consultation with the PCP, by two trained physicians, to all patients agreeing to participate. Of the original sample of 1716 subjects, 1467 agreed to participate (85.5% response rate). Verbal informed consent was obtained from the participants for publication of this research. All information was self-reported by the participants. No medical records or interviews by any PCPs were used as sources of data.

### Review instrument

We used SF-12 [2], in its validated Italian version [21], to measure HRQOL.

The eight health domains assessed by the SF-12 may be aggregated into two summary measures, the Physical Component Summary (PCS-12) and the Mental Component Summary (MCS-12), applying a scoring algorithm empirically derived from the data of a US general population survey [22]. As recommended, we have standardized our scores according to US norm data (mean score = 50; SD = 10), in order to facilitate cross-cultural comparison of results [6].

Chronic diseases were defined as one or more self-reported diagnoses for diseases of long duration and generally slow progression, such as hypertension, heart disease, diabetes, stroke, cancer, psychiatric disorders, osteoarthritis, respiratory diseases, etc. [23].

To assess smoking and alcohol consumption habits we used the questions and the definitions of current smoking, current and heavy drinking derived from the Behavioral Risk Factor Surveillance System questionnaire 2007 [24]. This questionnaire is currently adopted in a surveillance system on major behavioral risk factors in the Italian general population [25]. Excessive alcohol use, according to International Drinking Guidelines [26], was defined on the basis of either heavy drinking, binge drinking, or both.

Moreover, the questionnaire included information on patient’s socio-demographic characteristics and utilization of health services during the previous year, including information on number and main reasons for specialist visit, emergency access, and hospital admission.

The study protocol was approved by Ethics Committee of the “Mater Domini” Hospital of Catanzaro (Italy) (Prot. E.C. n. 127/2006).

### Statistical analysis

We performed univariate [t-Test and analysis of variance (ANOVA)] and multivariate (stepwise multiple linear regression) analysis to evaluate eventual significant differences of mean PCS-12 and MCS-12 according to various characteristics (demographics, presence of chronic diseases, behavioral risk factors, and utilization of health services). The two models were built since they allowed us to assess each of these differences independently of the other potential covariates. Therefore, modelling was only performed for adjustment of the differences and not for making predictions. All of the tests for significance were two sided and p-values ≤0.05 were considered statistically significant. The data were analyzed using the Stata software program, version 11 [27].

A detailed display of the covariates included in the univariate and multivariate analysis is reported in Table 1 and Table 2.

**Table 1:** **SF**-**12 summary scores according to selected characteristics of the study population**

**Characteristics**	**N (%)**	**PCS-12 Mean (±SD)**	**MCS-12 Mean (±SD)**
***Socio***-***demographic profile***			
**Sex**			
Male	660 (45)	46.8 (±10.4)	47.7 (±10.8)
Female	807 (55)	45.1 (±11.7)	42.6 (±11.7)
		t-test (1465) = 3.01, *p* = 0.003	t-test (1465) = 8.45, *p* < 0.001
**Age group, years**			
18-45	498 (33.9)	51.4 (±7.7)	46.3 (±11.4)
46-64	541 (36.9)	46.5 (±10)	44.9 (±11.6)
≥ 65	428 (29.2)	38.6 (±10.6)	43.2 (±11.7)
		F-test (2, 1464) = 214.95, *p* < 0.001	F-test (2, 1464) = 8.17, *p* < 0.001
**Marital status**			
Married	988 (67.3)	45.2 (±10.5)	45.2 (±11.5)
Other	479 (32.7)	47.2 (±11.1)	44.2 (±11.8)
		t-test (1465) = −3.34, *p* < 0.001	t-test (1465) = 1.50, *p* = 0.13
**Additional persons in the household**			
None	138 (9.4)	41.4 (±11.7)	43.5 (±11.2)
1	384 (26.2)	42.5 (±11.2)	44.3 (±12.2)
>1	945 (64.4)	47.9 (±9.9)	45.3 (±11.4)
		F-test (2, 1464) = 50.99, *p* < 0.001	F-test (2, 1464) = 2.23, *p* = 0.11
**Education level**			
No formal education	305 (20.8)	38.6 (±11.3)	42.9 (±12.6)
Primary school	309 (21)	43.4 (±10.5)	44 (±11.7)
Secondary school	588 (40.1)	49.2 (±9.1)	44.7 (±11.4)
University degree	265 (18.1)	49.8 (±8.5)	48.7 (±9.7)
		F-test (3, 1463) = 98.30, *p* < 0.001	F-test (3, 1463) = 13.49, *p* < 0.001
**Working activity**			
No	843 (57.5)	43.4 (±11.4)	43.6 (±12)
Yes	624 (42.5)	49.2 (±8.7)	46.6 (±10.9)
		t-test (1465) = −10.62, *p* < 0.001	t-test (1465) = −4.80, *p* < 0.001
***Utilization of health services during the previous year***			
**PCP accesses**			
<12	782 (53.3)	49.8 (±9)	47 (±11)
≥12	685 (46.7)	41.4 (±10.8)	42.5 (±11.9)
		t-test (1465) = 16.33, *p* < 0.001	t-test (1465) = 7.52, *p* < 0.001
**PCP medical visits**			
None	528 (36)	49.8 (±9.2)	47.3 (±11.4)
<12	590 (40.2)	46.5 (±10.3)	44.4 (±11.3)
≥12	349 (23.8)	38.9 (±10.4)	42.2 (±11.8)
		F-test (2, 1464) = 129.14, *p* < 0.001	F-test (2, 1464) = 21.99, *p* < 0.001
**Specialist visits in community health services**			
None	890 (60.7)	47.9 (±10.2)	46.1 (±11.5)
≥1	577 (39.3)	42.7 (±10.8)	43.1 (±11.5)
		t-test (1465) = 9.43, *p* < 0.001	t-test (1465) = 4.89, *p* < 0.001
**Private specialists visits**			
None	778 (53)	45.7 (±11)	45.6 (±11.5)
≥1	689 (47)	46.1 (±10.5)	44.1 (±11.5)
		t-test (1465) = −0.68, *p* = 0.50	t-test (1465) = 2.55, *p* = 0.011
**Emergency accesses**			
None	1129 (77)	46.5 (±10.6)	45.4 (±11.4)
≥1	338 (23)	43.7 (±10.9)	43.2 (±12.1)
		t-test (1465) = 4.28, *p* < 0.001	t-test (1465) = 3.11, *p* = 0.002
**Hospital admissions**			
None	1279 (87.2)	46.7 (±10.4)	45.3 (±11.6)
≥1	188 (12.8)	40.6 (±11.4)	42.1 (±11.5)
		t-test (1465) = 7.38, *p* < 0.001	t-test (1465) = 3.54, *p* < 0.001
***Behavioral risk factors and medical conditions***			
**Smoking habit**			
Never smoker	724 (49.3)	45.1 (±11.1)	44.6 (±12)
Ex-smoker	360 (24.6)	44.2 (±10.5)	45.9 (±10.4)
Current smoker	383 (26.1)	49 (±9.6)	44.6 (±12)
		F-test (2, 1464) = 10.03, *p* = 0.007	F-test (2, 1464) = 1.82, *p* = 0.162
**Excessive alcohol use**			
No	1346 (91.7)	45.7 (±10.7)	44.7 (±11.7)
Yes	121 (8.3)	47.9 (±10.8)	47.3 (±10.8)
		t-test (1465) = −2.13, *p* = 0.033	t-test (1465) = −2.41, *p* = 0.016
**Chronic diseases**			
0	569 (38.8)	52.3 (±6.9)	47.9 (±10.7)
1	478 (32.6)	45.3 (±10)	44.8 (±11.4)
2	270 (18.4)	39.5 (±10.2)	41.4 (±12)
≥3	150 (10.2)	34.9 (±9.7)	40.1 (±11.6)
		F-test (3, 1463) = 219.38, *p* < 0.001	F-test (3, 1463) = 31.17, *p* < 0.001
**Hypertension**			
No	973 (66.3)	48.4 (±9.9)	46.1(±11.5)
Yes	494 (33.7)	40.9 (±10.6)	42.5 (±11.6)
		t-test (1465) = 13.4, *p* < 0.001	t-test (1465) = 5.57, *p* < 0.001
**Diabetes**			
No	1300 (88.6)	46.7 (±10.5)	45.1 (±11.6)
Yes	167 (11.4)	39.2 (±10.7)	43.4 (±11.4)
		t-test (1465) = 8.79, *p* < 0.001	t-test (1465) = 1.71, *p* = 0.086
**Musculoskeletal problem**			
No	1303 (88.8)	47.2 (±10)	45.4(±11.4)
Yes	164 (11.2)	35.4 (±10.5)	41.1 (±12.7)
		t-test (1465) = 14.17, *p* < 0.001	t-test (1465) = 4.42, *p* < 0.001
**Heart disease**			
No	1352 (92.2)	46.6 (±10.6)	45.1(±11.6)
Yes	115 (7.8)	37.7 (±9.2)	42.5 (±11.3)
		t-test (1465) = 8.75, *p* < 0.001	t-test (1465) = 2.32, *p* = 0.02
**Gastrointestinal illness**			
No	1375 (93.7)	46.2 (±10.6)	45.2 (±11.5)
Yes	92 (6.3)	40.3 (±11.2)	39.7 (±11.9)
		t-test (1465) = 5.15, *p* < 0.001	t-test (1465) = 4.48, *p* < 0.001
**Asthma**/**COPD**			
No	1390 (94.7)	46.3 (±10.6)	44.9(±11.6)
Yes	77 (5.3)	38.9 (±10.3)	43.7 (±12.3)
		t-test (1465) = 5.91, *p* < 0.001	t-test (1465) = 0.93, *p* = 0.35
**Psychiatric disease**			
No	1431 (97.5)	46 (±10.7)	45.2 (±11.5)
Yes	36 (2.5)	41.9 (±10.7)	32.7 (±9.9)
		t-test (1465) = 2.26, *p* = 0.024	t-test (1465) = 6.44, *p* < 0.001

**Table 2:** Linear regression models results

	**PCS**-**12**				**MCS**-**12**			
	**F (19, 1447) = 53.81, p < 0.001, R2 = 0.41, Adjusted R2 = 0.41**				**F (17, 1449) = 15.00, p < 0.001, R2 = 0.15, Adjusted R2 = 0.14**			
	**COEFF**	**SE**	**t**	**P-value**	**COEFF**	**SE**	**t**	**P-value**
***Socio***-***demographic profile***								
Gender, (male as reference)								
Female	−1.50	0.47	−3.20	0.001	−4.58	0.59	−7.75	<0.001
Age, continuous	−0.14	0.02	−7.63	<0.001	-			
Marital status, (married as reference)								
Unmarried/widowed/divorced/separated	−0.54	0.49	−1.11	0.265	-			
Number in households, ordinal*	-				−0.90	0.49	−1.85	0.065
Education level, ordinal°	1.39	0.25	5.47	<0.001	0.89	0.32	2.78	0.005
Working activity, (unemployed as reference)								
Employed	-				-			
***Behavioral risk factors***								
Smoking habit, (never smoker as reference)								
Ex-smoker	−0.55	0.57	−0.98	0.327	-			
Current smoker	0.88	0.55	1.59	0.112	−2.29	0.68	−3.37	0.001
Alcohol abuse (non-excessive drinker as reference)								
Excessive drinker	−1.65	0.83	−2.00	0.045	1.63	1.07	1.53	0.127
***Chronic diseases***								
Hypertension (absence as reference)	−0.54	0.56	−0.97	0.333	−1.89	0.70	−2.70	0.007
Diabetes (absence as reference)	−1.87	0.73	−2.56	0.011	-			
Musculoskeletal problem (absence as reference)	−6.96	0.73	−9.47	<0.001	−1.48	0.94	−1.58	0.115
Heart disease (absence as reference)	−2.93	0.87	−3.37	0.001	−1.51	1.10	−2.70	0.173
Gastrointestinal illness (absence as reference)	−2.02	0.91	−2.22	0.026	−3.71	1.17	−3.15	0.002
Asthma/COPD (absence as reference)	−4.66	0.98	−4.74	<0.001	-			
Psychiatric disorders (absence as reference)	-				−10.11	1.84	−5.49	<0.001
***Utilization of health services during the previous year***								
PCP^§^ accesses, (<1 time per month as reference)								
≥1 time per month	−1.34	0.56	−2.40	0.017	−1.65	0.72	−2.31	0.021
PCP^§^ medical visits, ordinal^†^	−1.67	0.34	−4.87	<0.001	−0.86	0.44	−1.95	0.051
Specialist visits in community health services, (<1 time as reference)								
>1	−2.33	0.47	−4.99	<0.001	−1.45	0.61	−2.39	0.017
Private specialist visits, (<1 time as reference)								
≥1	−0.46	0.44	−1.04	0.300	−1.62	0.58	−2.81	0.005
Emergency accesses, (<1 time as reference)								
≥1	−1.18	0.55	−2.15	0.032	−1.06	0.71	−1.51	0.132
Hospital admissions, (<1 time as reference)								
≥1	−3.10	0.69	−4.53	<0.001	−1.51	0.89	−1.70	0.090

* Number in households: (none = 0, 1 = 1, >1 = 2); ° Education level: (no formal education = 1, primary school = 2, secondary school = 3, high school or higher = 4); ^§^ PCP = primary care physician; ^†^ PCP medical visits in the previous year: (none = 0, <1 time per month = 1, ≥1 time per month = 2).

## Results

Selected characteristics of the sample are reported in Table 1. Among the eligible respondents, the mean age was 52.3 years (range 18–87), 38.8% of respondents did not have any chronic disease, 32.6% had one, 18.4% had two and 10.2% had three or more chronic diseases. 33.7% of patients suffered from hypertension, 11.4% from diabetes, 11.2% from musculoskeletal problem, 7.8% from heart disease, 6.3% from chronic gastrointestinal illness and 5.3% from Asthma/COPD. Psychiatric disorders were reported by 2.5% of respondents.

The SF-12 items and PCS-12 and MCS-12 summary scores are presented in Table 3. 37.3% of the sample evaluated their health in general as poor or fair, and most of the items showed negative perceptions in percentages generally approaching or exceeding 40% of the sample, except for negative emotions (21.1%).

**Table 3:** **SF**-**12 items and summary scores**

Item description* (Health domain)	**Item response category frequencies (%)**					
	**Excellent**	**Very good**	**Good**	**Fair**	**Poor**	
1. General health perception (GH°)	6.5	16.6	39.6	27.3	10	
	**Yes, a lot**	**Yes, a little**	**Not limited**			
2. Limitations in moderate activities during a typical day (PF^§^)	11.2	26.4	62.4			
3. Limitations in climbing several flights of stairs during a typical day (PF^§^)	11.6	26.2	62.2			
	**Yes**	**No**				
4. Because accomplishment less of physical health (PR^†^)	37.3	62.7				
5. Limitation in work because of physical health (PR^†^)	35	65				
6. Because accomplishment less of emotional problems (ER^‡^)	42.9	57.1				
7. Because carefulness less of emotional problems (ER^‡^)	40	60				
	**Not at all**	**A little bit**	**Moderately**	**Quite a bit**	**Extremely**	
8. Interference of bodily pain with normal activities (BP^**^)	38.5	16.5	27.6	15	2.4	
	**All of the time**	**Most of the time**	**A good bit of the time**	**Some of the time**	**A little of the time**	**None of the time**
9. Calm and peaceful (MH°°)	11.7	26.8	13.7	29.9	13.5	4.4
10. Lot of Energy (V^§§^)	15.3	23.9	15	29.3	13.1	3.4
11. Downhearted and blue (MH°°)	4.3	9.1	7.7	30	30.1	18.8
12. Interference of physical or emotional problems with social activities (SF^††^)	1.8	7.7	-	26	25.7	38.8
**Summary scores**	**Median**	**Mean (±SD)**				
Physical Component Summary (PCS)	48.9	45.9 (±10.8)				
Mental Component Summary (MCS)	46.7	44.9 (±11.6)				

^*^ Items 4–12 investigate health conditions during the previous 4 weeks ; ^°^*GH* = General Health; ^§^*PF* = Physical Functioning; ^†^*PR* = Physical Role; ^‡^*ER* = Emotional Role; ^**^BP = Bodily Pain; ^°°^*MH* = Mental Health; ^§§^*V* = Vitality;^††^*SF* = Social Functioning.

The overall mean PCS-12 was 45.9 (SD ± 10.8; median = 48.9), whereas the overall mean MCS-12 was 44.9 (SD ± 11.6; median = 46.7).

Table 1 and Table 2 present the results of univariate and multivariate analysis. Univariate analysis highlighted significant differences by socio-demographic characteristics; indeed, PCS-12 and MCS-12 were significantly lower in females, elderly, unemployed and in less educated subjects. PCS-12 was significantly higher in the unmarried, but in contrast, was lower in subjects living alone. Also, MCS-12 was significantly higher in the excessive drinkers and PCS-12 in current smokers and excessive drinkers.

Worse PCS-12 and MCS-12 were reported by patients affected by at least one chronic disease, and the scores decreased with the increasing number of chronic diseases. Indeed, patients with three or more chronic diseases reported 17.4 and 7.8 points lower scores on PCS-12 and MCS-12 compared to subjects without chronic illnesses, respectively.

Finally, both MCS-12 and PCS-12 were significantly lower in subjects having higher health services utilization, such as PCP and specialist visits, emergency accesses, hospital admissions, etc.

When the multivariate analysis was performed, the results did not substantially change, with the exception of excessive alcohol use and current smoking patients that showed a significantly lower PCS-12 and MCS-12 scores, respectively.

## Discussion

The main result of our study is that it highlighted a poor perceived HRQOL of primary care patients in one of the poorest areas of Italy. Also, this study is one of the few that measured the HRQOL, analyzing both summary scores and SF-12 single items. The prevalence of subjects rating their health as unsatisfactory was one of the highest ever encountered in the literature [5,11,19,20,28-32], as well as at all SF-12-scales, particularly in Physical Functioning (PF), Emotional Role (ER), Bodily Pain (BP), and Social Functioning (SF).

Consistent with national ISTAT data [33], revealing that subjects living in the South presented a poorer self-perceived health status, we found that PCS-12 and MCS-12 were lower than Italian general population norms (mean PCS-12 = 50.4, mean MCS-12 = 49.8). Moreover, the comparisons with results reported by similar surveys show that our observed mean scores are generally considered substantially lower than those expected [5,16,29].

The reasons for the observed perceived results are complex and may be attributable to several factors. A possible reason may be cultural differences in values and reference levels, rather than true differences in health status [34]. However, as shown in previous research, objective differences in HRQOL across countries were frequently observed, and political and welfare variables have been associated to these differences, since countries with stronger social welfare orientations seem to impact positively on quality of life, while a poorer HRQOL was frequently observed in people living in Southern regions [35,36]. Likewise, it seems reasonable to hypothesize that in Italy the marked Northern–Southern divide, not only in economic development, but also in the distribution of public welfare resources [37], can have a possible role on HRQOL, although the assessment of this relation was not an aim of this survey.

In line with previous studies [8,9,14-20,30-32,35,38-41], our findings showed significant differences in HRQOL by socio-demographic characteristics and behavioral risk factors, with both lower scores reported by females, less educated patients, current smokers and excessive alcohol drinkers, whereas only lower PCS-12 was reported by older patients and only lower MCS-12 by separated or divorced patients.

As expected and according to other studies [5,11], we found that patient with chronic diseases reported significantly lower HRQOL and the decrements were larger in PCS-12 than in MCS-12. Patients with musculoskeletal problems and asthma/COPD showed lower PCS-12, while patients with hypertension and psychiatric disorders reported worse MCS-12. However, some of the observed results might reflect the combined influence of comorbid conditions, since a substantial percentage (28.6%) of primary care patients suffers from more than one chronic condition. Indeed, HRQOL was strongly poorer in patients affected by multimorbidities.

Poorer HRQOL was reported by patients with higher health services utilization, and this result is consistent with previous research [5,42]. Although it is well-known that health services utilization is related to many factors, such as patients’ preferences, awareness of their medical profile, availability of services and their expectations [42], and although our study was not designed to investigate utilization of health services according to HRQOL, it should be pointed out that our results seem to hypothesize that HRQOL may be used as a valuable tool for the estimation of health services needs [43,44]. In support to the potentials of the SF-12 in health services research, it may be mentioned the estimation of a preference measure of health derived from the SF-12 that has been proposed by Brazier and Roberts for the assessment of cost-effectiveness of health care intervention [45].

Our findings must be interpreted in the context of study’s limitations. First, as most research on this topic [5,8,11,14-20,28,29,31,32], our survey was performed as cross-sectional and it is well known that cross-sectional design does not allow any cause-effect relationship and poses many problems in relation to hypothesis testing since data on “risk factors” and “outcomes” are assessed at the same time. However, it was not our aim to draw conclusions on predictive relationships. Nonetheless, this study represents a useful way to determine the prevalence of poor HRQOL and, eventually, to identify HRQOL differences among subgroups disaggregated by demographics, presence of chronic diseases, behavioral risk factors, and utilization of health services, in order to target preventive interventions on those subjects that manifest poorer HRQOL. Second, data were based entirely on patients self-reporting; however, we do not think that method of data collection may represent a problem because self-reporting is the only way to collect subjective information about various domains of perceived health status. Third, as is the case of all questionnaire surveys, another limitation is the potential recall bias, especially in the elderly. However, recall bias was mitigated by having restricted recall within a specified period; in addition, the prevalence of elderly people in our study (29.2% aged ≥ 65 years and 3.1% aged ≥ 80 years) was similar or lower than in previous research on HRQOL [10,12,15,16,18,28,31,34], and the SF-12, being oriented more to perceptions rather than on objective health events, is particularly suited to the elderly.

## Conclusions

Our findings seem to indicate that primary care patients in Southern Italy have a poor HRQOL and this perception is even poorer in subgroups of the population, according to several sociodemographic, clinical characteristics, and behavioural risk factors. These results may have significant implications for health care policymakers, since they emphasize the need of developing effective and targeted strategies to improve HRQOL in Southern Italy, and provide support to monitor this process using the SF-12, that may be easily incorporated into the systematic evaluation of the patients in primary care settings. Further research is needed to replicate and validate these results on potential determinants of HRQOL through longitudinal studies, and to confirm the hypothesis that HRQOL may be used for the estimation of health services needs.

## Abbreviations

HRQOL: Health related quality of life; SF-12: Short-form-12 health survey; PCS-12: Physical component summary; MCS-12: Mental component summary; PCP: Primary care physician; GH: General health; PF: Physical functioning; PR: Physical role; ER: Emotional role; BP: Bodily Pain; MH: Mental health; V: Vitality; SF: Social functioning

## Competing interests

The authors declare that they have no competing interests.

## Authors’ contributions

BM collected the data, and contributed to the data analysis and interpretation and wrote the first draft of the article. PR, CP and AB contributed to conception and design of the study, collected the data, and contributed to the data analysis and interpretation. MP designed the study, was responsible for the data analysis and interpretation, have been involved in revising the manuscript and have given final approval of the version to be published. All authors read and approved the final manuscript.
